# Inhibiting tau-induced elevated nSMase2 activity and ceramides is therapeutic in murine Alzheimer’s disease

**DOI:** 10.21203/rs.3.rs-3131295/v1

**Published:** 2023-07-18

**Authors:** Carolyn Tallon, Benjamin J Bell, Medhinee M Malvankar, Pragney Deme, Carlos Nogueras-Ortiz, Erden Eren, Ajit G Thomas, Kristen R Hollinger, Arindom Pal, Maja Mustapic, Meixiang Huang, Kaleem Coleman, Tawnjerae R Joe, Rana Rais, Norman J Haughey, Dimitrios Kapogiannis, Barbara S Slusher

**Affiliations:** Johns Hopkins University; Johns Hopkins University; Johns Hopkins University; Johns Hopkins University; National Institute on Aging Laboratory of Clinical Investigation; National Institute on Aging Laboratory of Clinical Investigation; Johns Hopkins University; Johns Hopkins University; Johns Hopkins University; National Institute on Aging Laboratory of Clinical Investigation; Johns Hopkins University; Johns Hopkins University; Johns Hopkins University; Johns Hopkins University; Johns Hopkins University; National Institute on Aging Laboratory of Clinical Investigation; Johns Hopkins University

**Keywords:** Alzheimer’s disease, extracellular vesicles, neutral sphingomyelinase 2, tau, ceramide

## Abstract

**Background:**

Cognitive decline in Alzheimer’s disease (AD) is associated with prion-like tau propagation between neurons along synaptically connected networks, in part via extracellular vesicles (EV). EV biogenesis is triggered by ceramide enrichment at the plasma membrane from neutral sphingomyelinase2(nSMase2)-mediated cleavage of sphingomyelin. We report, for the first time, that tau expression triggers an elevation in brain ceramides and nSMase2 activity.

**Methods:**

To determine the therapeutic benefit of inhibiting this elevation, we evaluated the efficacy of PDDC, the first potent, selective, orally bioavailable, and brain-penetrable nSMase2 inhibitor, in the PS19 tau transgenic AD murine model. Changes in brain ceramide and sphingomyelin levels, Tau content, histopathology, and nSMase2 target engagement were monitored, as well as changes in the number of brain-derived EVs in plasma and their Tau content. Additionally, we evaluated the ability of PDDC to impede tau propagation in a murine model where an adeno-associated virus(AAV) encoding for P301L/S320F double mutant human tau was stereotaxically-injected unilaterally into the hippocampus and the contralateral transfer to the dentate gyrus was monitored.

**Results:**

Similar to human AD, PS19 mice exhibited increased brain ceramides and nSMase2 activity; both were completely normalized by PDDC treatment. PS19 mice exhibited elevated tau immunostaining, thinning of hippocampal neuronal cell layers, increased mossy fiber synaptophysin immunostaining, and glial activation, all pathologic features of human AD. PDDC treatment significantly attenuated these aberrant changes. Mouse plasma isolated from PDDC-treated PS19 mice exhibited reduced levels of neuron- and microglia-derived EVs, the former carrying lower phosphorylated Tau(pTau) levels, compared to untreated mice. In the AAV tau propagation model, PDDC normalized the tau-induced increase in brain ceramides and significantly decreased tau spreading to the contralateral side.

**Conclusions:**

PDDC is a first-in-class therapeutic candidate that normalizes elevated brain ceramides and nSMase2 activity leading to the slowing of tau spread in AD mice.

## INTRODUCTION

Alzheimer’s disease (AD) is the most common neurodegenerative disease, characterized by accumulation of amyloid plaques and tau-containing neurofibrillary tangles. Current treatment options are limited. Multiple therapeutic strategies are being pursued, with two monoclonal antibodies against amyloid-beta (Aβ) receiving FDA accelerated approvals based on reduction in brain amyloid plaques. Confirmatory trials focusing on clinical efficacy are ongoing and will be required for full approval (NCT05310071)(1), however, the clinical significance appears marginal(2). Deposits of hyperphosphorylated tau (pTau) constitute a second pathological hallmark of AD(3) and their co-occurrence with Aβ predicts cognitive decline(4). Tau can propagate between neuroanatomically connected regions in both a free form or via extracellular vesicles (EVs)(5, 6). Anti-tau antibodies have demonstrated the ability to sequester and degrade pTau aggregates(7), yet clinical trials to date have been unsuccessful(8, 9). EV-associated pTau in human AD has been characterized(10) and shown to have tau seeding capabilities(11), but, the therapeutic strategy of halting the spread of tau via EV inhibition remains unexplored.

The enzyme neutral sphingomyelinase 2 (nSMase2) cleaves sphingomyelin into ceramide, which enriches in the plasma membrane enhancing membrane curvature, resulting in EVs budding(12, 13). Studies from our laboratory(14, 15) and others(16, 17) using nSMase2 genetic knock-down as well as structurally distinct small molecule inhibitors have demonstrated that inhibition of nSMase2 halts the spread of pTau. Although these data are supportive of nSMase2 inhibition as a therapeutic approach, there are no available inhibitors suitable for clinical development. Employing high throughput screening followed by extensive medicinal chemistry(18, 19), our lab identified PDDC, the first nM potent, orally bioavailable, and brain penetrable small molecule nSMase2 inhibitor(18). We previously demonstrated that PDDC effectively reduces the plasma levels of brain-derived EVs following acute brain injury(20-25). Here, we build upon these results and evaluate PDDC’s potential to attenuate tau propagation and disease progression in PS19 tau transgenic mice as well as in an AAV-mediated human tau propagation model.

Herein we show, for the first time, that mutant tau expression is associated with increased brain ceramides in mice, similar to what has been reported in AD patients(20-25), as well as elevated brain nSMase2 activity. PDDC normalizes these elevations resulting in reduced pTau propagation and disease progression. In PS19 mice, PDDC also reduced hippocampal pTau, prevented thinning of the pyramidal and granule cell layer, and reduced glial activation, pathologies described in AD patients(26-28). PDDC-treated PS19 mice had reduced concentrations of neuron- and microglia-derived EVs in their plasma, with neuron-derived EVs showing decreased pTau levels. These data provide compelling evidence to support the clinical translation of PDDC in AD.

## MATERIALS AND METHODS

### Study Design

This study examined the effect of tau expression on nSMase2 enzymatic activity and ceramide production as well as the therapeutic efficacy of the nSMase2 inhibitor PDDC in mouse models of AD. We utilized two distinct AD mouse models: PS19 transgenic mice and an AAV-hTau(P301L/S320F) propagation model. Animals were randomly assigned to either vehicle or drug groups with an equal number of male and female in each. All data were acquired in a blinded manner with a number assigned to each animal unrelated to their treatment status. Where possible, the experimenter dosing the animals was different from the experimenter carrying out the data acquisition and statistical analysis to maintain the blind. Sample sizes were determined using power calculations based on previously observed statistically significant differences to generate at least 90% power with fve extra animals per group to account for any premature animal losses.

### Primary neuronal cell culture

Primary hippocampal neurons were isolated from day 18 embryos of Sprague-Dawley rats acquired from Jackson Laboratories (Bar Harbor, ME), as previously described(29-31). Briefly, hippocampal tissue was dissected, gently titurated with trypsinization in calcium-free Hank’s balanced salt solution (HBSS, calcium, magnesium, and phenol red free; Corning Inc., Manassass, VA) and cells were resuspended in Neurobasal media (Gibco, Waltham, MA) supplemented with B27 (Gibco), 1% antibiotic/antimitotic solution (Gibco), 10% FBS (Sigma, St. Louis, MO), Hepes (4.8mM, Sigma) and L-glutamine (1.2mM, Sigma). For imaging studies, cells were plated on 12mm glass coverslips coated with polyethyleneimine (PEI, Sigma) at a density of 70,000cells/well. For nSMase2 activity and ceramide analyses, cells were directly plated on PEI coated 12-well cell culture plates at a density of 700,000cells/well. After 4 days in vitro (DIV), cells were transduced with either pAAV1-CAG-GFP (Addgene, Watertown, MA) or an AAV1-serotype viral particles packaged with the hTau vector CBA-hTau24(P301L)(S320F)-WPRE (kindly provided by the Chakrabarty lab (University of Florida, Gainesville, FL))(32). Cells were transduced at a multiplicity of infection (MOI) of 50,000. At 8 DIV, cells were harvested for nSMase2 activity and ceramide assessments or prepped for imaging. Cells for nSMase2 activity were washed twice with ice-cold DMEM/F12 containing HEPES and without phenol red (ThermoFisher) and incubated with mammalian protein extraction reagent (ThermoFisher) containing 1X HALT protease inhibitor without EDTA (ThermoFisher), with shaking, for 10min at 4°C and for another 5min at RT. Final cell detachment was carried out using cell scrapers and sonicated (three 15s pulses on ice). The resulting lysates were assayed for both nSMase2 activity and total protein content as detailed below. Cells harvested for ceramide assessments were washed 3x on ice with 1X PBS prior to gently scraping with a cell scraper, centrifugation at 300*×g* for 5min at 4°C, freezing the pellet in liquid nitrogen, and storing at −80°C. Cells for imaging were fixed with ice cold 4% paraformaldehyde (PFA, Electron Microscopy Sciences) for 10min and washed 3x in 1X PBS followed by immediate staining.

### Animal studies and PDDC dosing

All animal care and experimental procedures complied with the National Institutes of Health guidelines on animal care and were approved by the Johns Hopkins University Institutional Animal Care and Use Committee. Mice were housed in a temperature and humidity-controlled environment under a 14-h light, 10-h dark cycle. Food and water were available *ad libitum*. Animals acclimated to the facility for at least 7 days after arrival, prior to any experimentation. PDDC was synthesized in our laboratory as previously described(18, 19), before being formulated into an OpenStandard Diet (15 kcal% mouse chow) at an approximate 100mg/kg daily dose and treated as previously described(33). PS19 breeder mice were purchased from Jackson Laboratories (Bar Harbor, ME; strain #008169) and bred in-house to generate appropriately sized litters. Non-carrier littermates were deemed WT controls. Dosing for PS19 animals was initiated at 4 months of age, prior to overt pathology and symptom onset(34), and continued until the animals were 9 months of age. For the AAV-hTau mice, C57BL6/J mice were purchased from Jackson Labs (strain #000664) and stereotaxically injected at 10 weeks of age and then dosed for 6 weeks. Weekly body weights were measured for all studies. Equal male and female animals were enrolled. No significant differences between males and females were observed so the groups were combined.

At the end of the study, animals were euthanized using an overdose of isoflurane. The chest cavity was opened up and blood was collected via cardiac puncture into cold EDTA-coated BD microtainers (Franklin Lakes, NJ). For histological assessments, the animals were cardiac perfused with 1Xice cold PBS followed by 2% paraformaldehyde (PFA; Electron Microscopy Sciences). For all other experiments, mice were cardiac perfused with PBS only. Tissue was collected following perfusion and the assays were performed as described below.

### PDDC in vivo pharmacokinetics (PK) and bioanalysis

12 mice (6 male/6 female) were enrolled in a PK study with n = 3/time point and the brain and plasma drug levels were quantified at 4 timepoints throughout the 24h day (00:00, 07:00, 12:00, and 19:00). Times were chosen based on the 14h light-10h dark cycle where 7:00 is when the lights come on and 19:00 was 2h before lights went off. Plasma was isolated from fresh whole blood by centrifugation at 500*×g* for 15min and stored at − 80°C until LC/MS/MS bioanalysis. Whole brains were harvested following blood collection and cut into hemispheres before freezing in liquid nitrogen and stored at − 80°C.

The bioanalysis was carried out as previously described(18, 33). Briefly, protein precipitation using acetonitrile (Sigma-Aldrich, St. Louis, MO*)(*100% v/v) containing the internal standard (losartan 500nM; Tocris, Minneapolis, MN) was used to extract PDDC standards and samples from plasma and brain prior to vortexing and centrifugation at 10,000*×g*. The supernatant was diluted 1:1 with water and then analyzed via LC/MS/MS. Plasma (nmol/ml) and tissue (nmol/g) concentrations were determined and plots of mean plasma concentration versus time were constructed. Phoenix WinNonlin version 7.0 (Certara USA, Inc., Princeton, NJ) was used to quantify exposures (AUC_0–t_) using non-compartmental analysis modules.

### nSMase2 enzymatic activity assay

nSMase2 enzymatic activity was assessed as previously described(15, 33, 35, 36). nSMase2 activity measurements were initiated upon the addition of sphingomyelin (SM) and coupling enzymes, in the Amplex Red system (25 μl), and SM hydrolysis carried out in total reaction volumes of 50 μl in 384-well microplates for 3h at 37°C. At the end of the reaction period, the relative fluorescence units were measured at Ex 530nm, Em 590nm. Total protein measurements were carried out as per manufacturer’s instructions using PierceTM 660nm Protein Assay Reagent (ThermoFisher) and data presented as RFU/mg/h.

### Brain lipid extraction and LC-ESI-MS/MS ceramide quantification

Lipid extractions of hippocampal cells and brain tissue from a single hemisphere (for PS19) or micro-dissected hippocampi and cortex (for AAV-hTau) were carried out using a modified Bligh & Dyer method(37) as previously described(38). Tissue samples were weighed and homogenized in water (10×) before adding 3× methanol containing a 1.3μg/mL internal standard of ceramide (d18:1/12:0*)(*Avanti Polar Lipids, Alabaster, AL, USA))(39) followed by an addition of 4× chloroform. Organic layers containing crude lipid extracts were collected following clear phase separation, before being dried in a nitrogen evaporator (Organomation, Berlin, MA, USA) and stored at − 80°C. Prior to analysis, pure methanol was used to resuspend the dried extracts. A Shimadzu ultra-fast liquid chromatography (UFLC) system (Shimadzu, Nakagyo-ku, Kyoto, Japan) coupled to a hybrid triple quadrupole LIT (linear ion trap) mass spectrometer 4000 QTRAP system equipped with Turbo Ion Spray (SCIEX, Foster City, CA, USA) with an ULTRA HPLC In-Line Filter (0.5μm Depth Filter×0.004 in ID*)(*Phenomenex, Torrance, CA, USA) was used to separate ceramides on a C18 reverse-phase column (2.6μm, 50×2.1mm). The lipids were ionized using positive electrospray ionization (ESI, +ve) and individual ceramide species were detected by multiple reaction monitoring (MRM) with instrument conditions and HPLC parameters previously described(40). Quality control (QC) samples were injected in every 10 injections. Eight-point calibration curves (0.1–1000ng/mL) were constructed by plotting area under the curve (AUC) for each ceramide calibration standard d18:1/C16:0, d18:1/C18:0, d18:1/C20:0, d18:1/C22:0, d18:1/C24:0 (Avanti polar lipids, Alabaster, AL, USA) with correlation coefficients >0.999. Identified ceramide concentrations were calculated by fitting them to these standard curves based on acyl chain length. Instrument control and data acquisition were performed by using Analyst (version 1.4.2, SCIEX Inc. Thornhill, ON, Canada) and data analysis were completed using MultiQuant software (version 2.0, SCIEX).

### Hippocampal tau isolation and western blotting

Left and right hippocampus were micro-dissected from whole brains following PBS perfusion, weighed, and then snap frozen on dry ice. Hippocampus tissue was mechanically homogenized in ten volumes of ice cold 1X RIPA buffer (Thermo Fisher) with Pierce Protease and Phosphatase Inhibitor Mini Tablets (Thermo Fisher Scientific, Waltham, MA) followed by brief sonication. The lysate was centrifuged for 15min at 10,000*×g* at 4°C and the supernatant was collected, frozen on dry ice, and stored at −80°C. Sarkosyl soluble and insoluble isolation was carried out based on a modified version of Sahara and Kimura (2018)(41). Briefly, hippocampus tissue was mechanically homogenized in ten volumes of ice cold 2X TBS buffer (Thermo Fisher Scientific) with protease and phosphatase inhibitor cocktail prior to being centrifuged at 27,000*×g* at 4°C for 20min. The pellet was resuspended in five volumes of ice-cold high salt/sucrose buffer and centrifuged at 27,000*×g* at 4°C for 20 min. The supernatant was then adjusted to 1% sarkosyl and incubated on a shaker for 1h at 37°C before ultracentrifugation at 150,000*×g* at 4°C for 1h. The sarkosyl soluble supernatant was removed and frozen on dry ice to be stored at −80°C. The sarkosyl insoluble pellet was resuspended in 0.5 volume of 1X TE buffer (Thermo Fisher Scientific) and frozen on dry ice to be stored at −80°C. All western blots were run in a similar manner. Equal volumes of samples were loaded onto a NuPAGE 4–12% bis-tris protein gel (Invitrogen) and transferred on to an PVDF membrane using an iBlot2 Gel Transfer Device (Life Technologies). Total protein stain was performed for loading controls using Revert 700 total protein stain (LI-COR). An HRP-conjugated GAPDH antibody was also used as a loading control where applicable. Blots were blocked with EveryBlot blocking buffer (Bio-Rad) and stained overnight at 4°C for total tau (Tau 46; Santa Cruz Biotechnology, #sc-32274) and Thr181 phosphotau (D9F4G; Cell Signaling Technologies, #12885S). Appropriate HRP-conjugated secondary antibodies were used. Blots were incubated briefly with Clarity ECL substrate (Bio-Rad). All blots were imaged using the Bio-Rad ChemiDoc MP imager. Analysis was done using raw TIFF files in ImageJ. Mean pixel intensity was measured for each band and normalized to total protein or GAPDG intensity levels. To compare across blots, each blot was also normalized to the average value of the vehicle intensity.

### Immunofluorescence staining

*Fixed primary neuronal cells* were permeabilized using 0.1% Triton X-100 in 1X PBS (0.1% PBST) for 10 min at room temperature (RT) before blocking with 5% normal goat serum in 0.1% PBST for 1h at RT. Primary antibody to phosphotau Thr181 (pThr181-Tau, Cell Signaling Technology) was incubated overnight at 4°C followed by the appropriate secondary antibody for 1h at RT. Neurons were stained using Alexa Fluor^®^ 647 conjugated Anti-NeuN antibody (Abcam, #ab190565) for 2h at RT. Nuclei were then stained with Hoechst 33342 (Invitrogen, #H3570) before mounting with ProLong Glass antifade mountant (Invitrogen).

*Brain tissues* were prepped for immunofluorescence staining as previously described(15, 42). Briefly, following PFA perfusion, whole brains were dissected out and post-fixed overnight at 4°C in 2% PFA before being transferred to 15% then 30% sucrose, each overnight at 4°C. Brains were then frozen in TissueTek O.C.T. (Sakura FineTek USA, Inc., Torrence, CA, USA) and sectioned on a cryostat (Microm HM 505E, International Medical Equipment, MI, USA) at 20μm thickness. Sections were permeabilized and blocked followed by primary antibody (pThr181-Tau, Cell Signaling Technology; GFAR Abcam, #ab4674; Iba1, Fujifilm Wako Chemicals, #019-19741; Synaptophysin, SinoBiological, #100298-T40) incubation overnight at 4°C. Sections were then stained with appropriate secondary antibodies for 1h at room temperature. Neurons were then stained with Alexa Fluor^®^ 647-conjugated Anti-NeuN antibody (Abcam, #ab190565) for 2h at RT. Nuclei were stained with Hoechst 33342 (Invitrogen, #H3570) before coverslipping with ProLong Glass antifade mountant. All slides grouped together for MFI assessments were stained within the same batch of slides to minimize possible differences in antibody amounts and incubation times.

All images were taken on a LSM 800 confocal microscope (Zeiss) using identical imaging parameters for all images acquired. Images were acquired by focusing to the center of the section where the signal of interest was at a maximal intensity, with the brightest slide used to set the imaging parameters. Both hippocampi from 3–5 sections were imaged per animal and 3–6 images were acquired per hippocampal section (CA1, CA3, and DG, where applicable). Values from the images of both hippocampi per section were averaged and reported. All image analysis was done using Zen Blue imaging software (Zeiss).

### Single cell mean fluorescence intensity (MFI) quantification

Single cell MFI was determined using images stained with pThr181-Tau, NeuN, and Hoechst 33342 and imaged with a 40X objective. Tau positive neurons were determined based on triple staining of tau, NeuN, and nuclei in order to ensure cells counted were imaged at a similar level and differences in intensity were not due to different imaging planes. Cells deemed “tau+” then had their cell bodies intricately traced, stopping at the axonal hilus as it was not possible to include axons and dendrites in the tracing. The MFI of the tau signal was recorded for each cell and an average of each individual cell per section was determined and recorded.

### Pyramidal and granular cell layer thickness

Cell layer thickness was determined using images stained with NeuN and Hoechst 33342 with a 40X objective as previously reported(43). The thickness of the NeuN cell layer in the CA1 and DG regions was determined by drawing a line perpendicular to the cell layer at three points along the layer in each image, taking the thickest, thinnest and middle of the section. The three values were then averaged and the values for each section were then averaged and reported as a replicate. The CA3 region was not evaluated as it was more subject to slight variations in the plane of section and was highly variable.

### Synaptophysin fluorescence intensity quantification

To quantify synaptic loss previously observed in PS19 mice(34), we measured the MFI intensity of synaptophysin staining in the Mossy fiber layer of the CA3. Images were acquired with identical parameters using a 20X objective and three images per hippocampus were taken from three sections per mouse. The Mossy fiber layer was traced and the MFI was recorded from each image. The values of all images per section were averaged and reported as a replicate.

### Iba1 and GFAP intensity quantification

Sections were stained with Iba1, GFAR and Hoechst 33342 and imaged with a 20X objective with identical parameters. 8 images per section from 3 sections per animal were obtained with identical parameters in the stratum radiatum, stratum moleculaire, and hilus regions around the CA1, CA3 and dentate gyrus regions. The MFI of the entire field of view was recorded and the average of all images per section was reported as a replicate.

### Plasma nEV isolation, quantification and characterization

Mouse plasma nEV isolation was carried out as previously described(44). Briefly, plasma samples were defibrinated with Thrombin (System Biosciences, Mountainview, CA, USA) for 30min at RT and total EVs isolated via size exclusion chromatography (SmartSEC, System Biosciences). NEVs were isolated from total EVs via immunocapture against L1CAM/CD171 (clone 5G3). Protease and phosphatase inhibitors were included in multiple steps. Intact EVs were used for determination of particle concentration and diameter using nanoparticle tracking analysis (NTA)(Nanosight NS500; Malvern, Amesbury, UK). To confirm the isolation of *bona fide* EVs via L1CAM immunocapture, 30μL of intact L1CAM + nEVs were subjected to ExoView^™^ for the fluorescent detection of canonical EV markers CD9, CD63 and CD81 (NanoView Biosciences, #EV-TETRA-M2). Additionally, nEVs were lysed with protein extraction solution and the protein concentration was determined using the Bradford protein assay (Bio-Rad, Hercules, CA, USA). The pThr181-tau content was quantified in duplicate using the Human Tau pT181 ProQuantum Immunoassay Kit as per the manufacturer’s protocol (Invitrogen) at a dilution of 1:4. Samples were read on a qPCR equipment (StepOnePlus, Applied Byosystems). All Ct values were below 35. The LOD was found to be 0.0847pg/mL. To avoid excluding WT samples from the analysis, since they lack human tau, samples read below the limit of detection were set at a value of ½ LOD.

Nanoscale multiplex flow cytometry analysis (FCA) was carried out as previously described with slight modifications(45). Intact L1CAM + nEVs or total EVs isolated via SEC were diluted to with 1X-PBS and incubated with an equal volume of 40 μM blue succinimidyl ester (BSE; Thermo Fisher Scientific, #C34568) for 90min at 37°C. Excess BSE was removed via ultrafiltration (100kDa cutoff; Millipore Sigma, #C7719) bringing the final retentate volume to 1mL with 1X-PBS incubated with 100μL of Capto Core 400 beads (Cytiva, #17372401) for 30mins at RT with gentle rotation mixing. The EV supernatant was then incubated with a mouse-specific Fc receptor blocker reagent (Miltenyi, #130-092-575) for 30mins at RT with gentle rotation and labeled with fluorescent antibodies (each at 0.2ng/μL; PE-anti-Syntenin-1, Abcam, #210837; PE-anti-pSer262-Tau, Thermo Fisher Scientific, #44-750G; APC-anti-β-III-tubulin, Biolegend, #801219; APC-Iba-1, Abcam, #5076) in 0.05% tween-20 for partial membrane permeabilization. Labeled EVs were detected with a CytoFLEX LX flow cytometer (Beckman Coulter) using 405nm fluorescence triggering and analyzed with CytExpert software v2.3.0.84 (Beckman Coulter). For fluorescence detection, we used a 660/10 bandpass filter for APC, and 585/42 for PE, with gain voltage not exceeding 1500V. The instrument was aligned using FITC-tagged beads with sizes ranging from 100 to 1300nm (100nm beads, #834, Bangs Laboratories; 130–1300nm beads, #NFPPS-52-4K and #NFPPS-0152-5, Spherotech). Samples were diluted with 1X-PBS to control the abort rate below 1% without exceeding 200 events/second rate to avoid coincident detection of events and analyzed for 3mins.

### AAV-hTau(P301L/S320F) stereotaxic injection model

The rapid tau propagation model was performed as previously described(15). The same AAV-hTau vector used in the cell culture experiments was used for the animal injections. A modified stereotaxic surgical method was used(16, 32), where mice had the AAV vector injected into their left dorsal hippocampus near the CA3 region using a stereotaxic apparatus (Stoelting, Wood Dale, IL, USA) and a pulled glass capillary needle (tip diameter < 50μm) at the coordinates AP-2.3, ML-2.1, and DV-2.2. 5×10^9^ viral particles in < 250nL PBS were injected using a digital nanoinjector (Stoelting) attached to a mineral oil-filled 5uL gas-tight syringe (Hamilton) over 5min and the syringe was left in place for an additional 5min. Afterwards, the syringe was removed and the incision closed with cyanoacrylate glue (Vetbond, 3M) and the mouse provided ketoprofen analgesia and monitored for distress over 48h. Mice were given 2 days of recovery prior to treatment initiation and were treated for 6 weeks.

### Contralateral tau mean fluorescence intensity quantification

Sections from the AAV-hTau mice were analyzed as previously described(15). Following staining for NeuN, phosphor tau, and Hoechst 33342, 2 images each from both the left and right dorsal dentate gyrus were acquired on an LSM 800 confocal microscope (Zeiss). The two images per section were averaged and each section was treated as a replicate. The ratio of the contralateral to ipsilateral MFI per section was calculated to account for injection variability. Animals with improper injection sites were excluded from analysis.

### Statistical Analysis

All statistical analysis was done using GraphPad Prism 9 (GraphPad Software, LLC, San Diego, CA, USA). Comparisons made between two normally distributed groups utilized a two-tailed, unpaired student’s t-test. Comparisons made between two non-parametric groups utilized a Mann-Whitney U test. Comparisons made between three or more groups utilized a one-way ANOVA with Tukey’s multiple comparison. Results were considered statistically significant when p < 0.05.

## RESULTS

### Mutant tau expression in cultured neurons increases nSMase2 activity and ceramides

**Figure 1:**
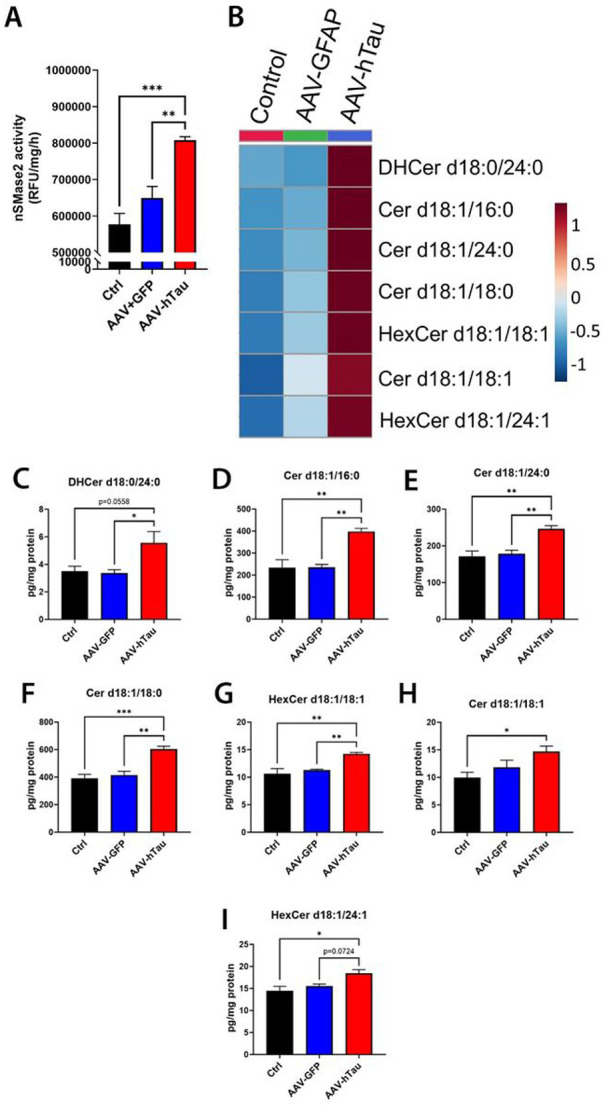
Mutant tau expression induces a significant increase in nSMase2 activity and ceramide levels in cultured neurons. **A**) nSMase2 activity from untransduced control (Ctrl), AAV-GFP transduced, and AAV-hTau(P301L/S320F) transduced cells. **B**) Heat map of the significantly elevated ceramide species in AAV-hTau(P301L/S320F) transduced cells compared to either control or AAV-GFP transduced cells. Colors represent fold-change in relative-abundance compared to untransduced control cell levels. **C-I**) Individual levels of the altered ceramides. N=4/group. Bars represent mean±SEM. *p<0.05, **p<0.01, ***p<0.001. One-way ANOVA with Tukey’s multiple comparison.

We examined the effect of mutant tau expression in neurons on nSMase2 activity and ceramides in primary rat hippocampal neurons. We observed GFP expression in control AAV-GFP transduced cells and pTau-Thr181 staining in AAV-hTau transduced cells (Fig S1, middle and bottom rows, respectively). No fluorescent signal was observed in the untransduced controls (Fig S1, top row). AAV-hTau cells exhibited significantly elevated nSMase2 activity compared with untransduced controls (Fig. 1A; p = 0.00036) and AAV-GFP controls (p = 0.0048). Of the 22 ceramide species detected, 7 were found to be significantly elevated in AAV-hTau transduced cells compared to untransduced and/or AAV-GFP transduced cells (Fig. 1B-I and Table S1). All other forms of ceramides remained unchanged.

### Oral PDDC provides sustained drug levels in the brain that inhibit nSMase2 activity and reduce ceramide levels

**Figure 2:**
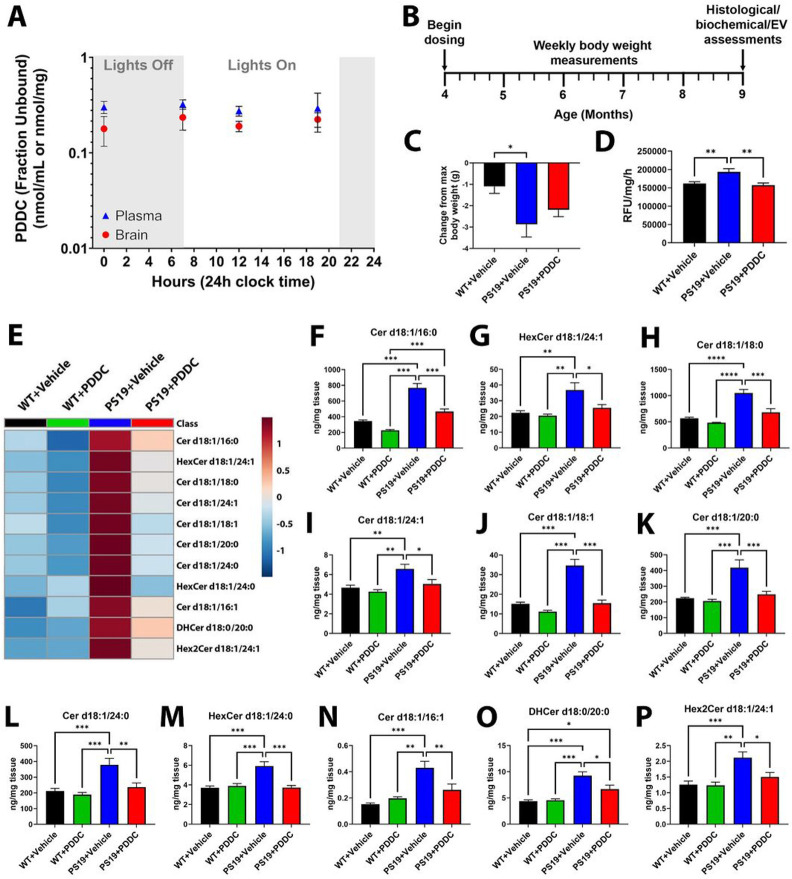
Brain ceramides are robustly elevated in PS19 mice and are normalized with PDDC treatment. **A**) Plasma and brain levels of PDDC measured over 24h following 4 weeks of dosing. N=3/group/time point. Points represent mean±SEM. **B**) Dosing schematic. **C**) Percent change of body weight at the time of sacrifice from the maximum body weight over a 5-month dosing period from WT+Vehicle, PS19+Vehicle, and PS19+PDDC groups. N=16-20. **D**) Quantification of hippocampal nSMase2 activity in WT+Vehicle, PS19+Vehicle, and PS19+PDDC mice. N=8-10/group. **E**) Heatmap showing the ceramide species significantly reduced in PDDC-treated PS19 mice compared to vehicle-treated PS19 mice(p<0.05). Colors represent relative abundance of each ceramide. **F-P**) Cortical ceramide levels in WT and PS19 mice chronically treated with vehicle or PDDC. N=6-11/group. Bars represent mean±SEM. *p<0.05. **p<0.01. ***p<0.001. One-way ANOVA with Tukey’s multiple comparison.

We next examined the effect of PDDC on brain nSMase2 activity and ceramide levels in PS19 mice. After 4 weeks on PDDC-containing chow (approximately 3mg PDDC ingested daily), PS19 mice exhibited sustained plasma and brain levels of PDDC at free concentrations (fraction unbound) around the IC_50_ of PDDC for nSMase2 (300nM)(Fig. 2A). PDDC chow was fed 5 days/week to PS19 mice starting at 4-months-of-age, and continued until 9-months-of-age (Fig. 2B). After 5-months of treatment, relative to their peak weight, vehicle-treated PS19 mice lost more weight compared to WT controls (Fig. 2C, p = 0.018). PDDC-treated mice showed a trend toward reduced weight loss, however, were not different from vehicle-treated PS19 mice (Fig. 2C). Additionally, physical characteristics and behaviors were not different from WT mice as determined with a modified SHIRPA test (Table S2). In open field testing, both PDDC and vehicle-treated PS19 mice showed more ambulatory movement versus WT mice, as has been previously described(46). There were no differences between vehicle or PDDC treated mice in overall distance travelled, fine movement, rearing, or center/periphery ratio (Fig S2). In all experimental groups, plasma clinical chemistry parameters to assess liver and kidney toxicity were within the normal ranges observed in our facility and reported by Jackson Laboratories(47-49), Charles River(50), or Taconic(51) (Table S3).

Similar to cells transduced with AAV-hTau, we found that hippocampal nSMase2 activity in PS19 mice was significantly elevated compared to WT mice at 9-months of age (Fig. 2D, p = 0.0057). PDDC treatment completely normalized this elevation, demonstrating clear target engagement (Fig. 2D, vs PS19 + vehicle p = 0.003). Of the 46 different forms of ceramide detected, 11 were increased in vehicle-treated PS19 mice compared to vehicle-treated WT mice (Fig. 2E-P; Table S4, p values found in table). All ceramides elevated in PS19 mice were reduced with PDDC to WT + vehicle concentrations. PDDC did not alter ceramide levels in WT mice.

### PDDC treatment reduces tau pathology in PS19 mice

**Figure 3:**
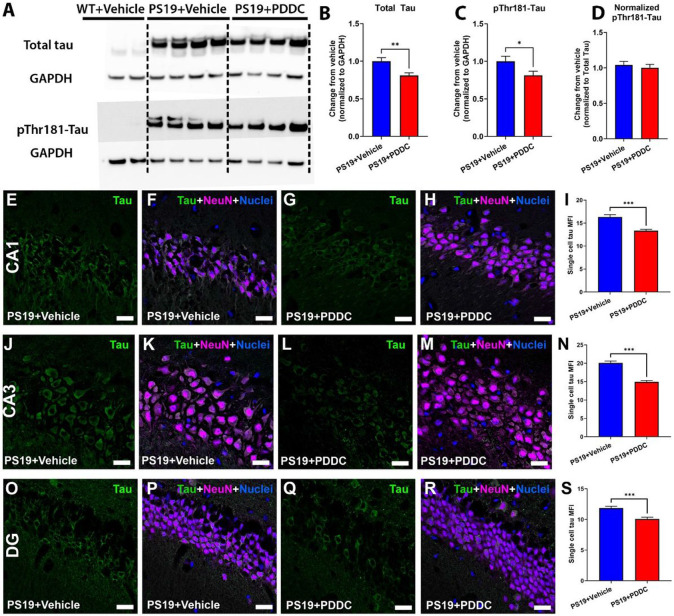
PDDC treatment reduces hippocampal tau levels in PS19 mice. **A**) Representative Western blots from micro-dissected hippocampal tissue showing total human tau (upper blot) and pThr181-Tau (lower blot). GAPDH shown as a loading control. **B**) Quantification of Western blots for total tau. **C**) Quantification of Western blots for pThr181-Tau. **D**) pThr181-Tau level normalized to total tau. N=11-12/group. Representative images showing pThr181-Tau staining (green) and neuronal staining (magenta) from vehicle and PDDC treated PS19 mice at the CA1 (**E-H**), CA3 (**J-M**) and dentate gyrus (DG, **O-R**). Single cell mean fluorescence intensity (MFI) from the CA1 (**I**), CA3 (**N**), and DG (**S**). Nuclei shown in blue. N=120cells/group from 4 mice/group. Bars represent mean±SEM. *p<0.05, **p<0.01, ***p<0.001. Scale bar, 20μm. Gamma and brightness adjusted equally for all images presented. All graphs, unpaired two-tailed t-test.

We next assessed whether PDDC altered tau pathology in PS19 mice. Total tau in the hippocampus of PDDC-treated PS19 mice was reduced compared with vehicle-treated mice (Fig. 3A-B, p = 0.0041). No human tau was detected in WT animals (Fig. 3A). Although pThr181-Tau was also reduced in PDDC-treated mice (Fig. 3C, p = 0.047) this difference was lost with total tau normalization (Fig. 3D), suggesting PDDC does not impact the phosphorylation of tau at Thr181. PDDC also did not affect the balance of Sarkosyl soluble and insoluble tau fractions (Fig S3). We further assessed tau levels by quantifying single cell tau fluorescence intensity in the hippocampus (Fig. 3E-S). Tau fluorescence intensity in individual neurons were reduced in the CA1 (Fig. 3E-I, p = 9.56×10^−7^), CA3 (Fig. 3J-N, p = 3.49×10^−10^), and DG (Fig. 3O-S, p = 1.57×10^−4^) regions of the hippocampus of PDDC-treated PS19 mice compared with vehicle-treated mice.

### PDDC treatment reduces hippocampal cell layer thinning and mossy fiber synaptophysin loss in PS19 mice

**Figure 4:**
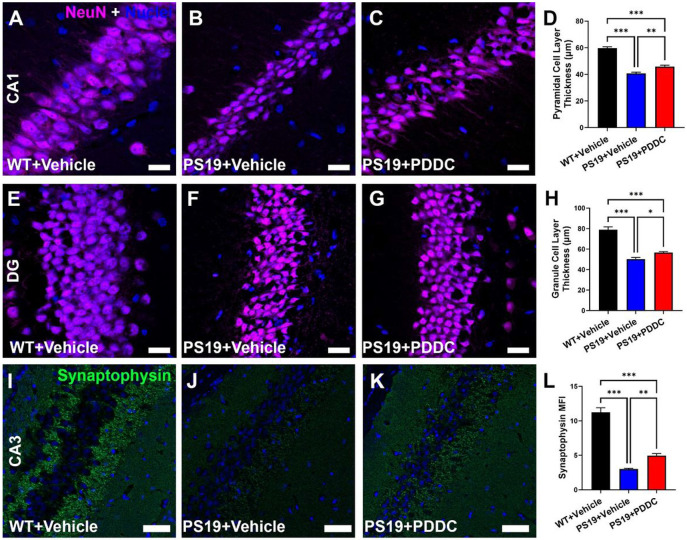
PDDC reduces hippocampal cell layer thinning and mossy fiber synaptophysin loss in PS19 mice. **A-H**) Pyramidal cell layer thickness from the CA1 region (**A-D**) and granule cell layer thickness from the dentate gyrus (DG, **E-H**). Representative images showing NeuN neuronal staining (magenta) from WT (**A, E**), vehicle-treated PS19 (**B, F**) and PDDC-treated PS19 (**C, G**) mice. Nuclei shown in blue. Scale bar, 20μm. **D, H**) Quantification of neuronal cell density counts from CA1 (**D**) and DG (**H**). N=122-141 images/group from 7-8 mice/group. **I-L**) Synaptophysin staining (green) of the mossy fiber layer in the CA3 from WT (**I**), vehicle-treated PS19 (**J**), and PDDC-treated PS19 (**K**) mice. Scale bar, 50μm. **L**) Quantification of the mean fluorescence intensity (MFI) of synaptophysin staining in the mossy fiber layer. Bars represent mean±SEM. *p<0.05, **p<0.01, ***p<0.001. N=45-54 images/group from 5-6 mice/group. One-way ANOVA with Tukey’s multiple comparison.

We observed a thinning in the pyramidal cell layer of the CA1 region (Fig. 4A-B, D, p = 1.99×10^−11^) and granule cell layer of the dentate gyrus (Fig. 4E-F, H, p = 1.79×10^−11^) in the PS19 mice versus WT mice. PDDC reduced this thinning (Fig. 4B-D and Fig. 4F-H, p = 0.00127 and p = 0.0118, respectively). Synaptophysin staining of mossy fibers in the CA3 region was reduced in PS19 mice compared to WT mice (Fig. 4I-J, L, p = 2.08×10^−12^). PDDC increased the synaptophysin staining (Fig. 4I, K-L) compared to vehicle-treated PS19 mice (Fig. 4J-L, p = 0.00923).

### PDDC treatment reduces glial activation in PS19 mice

**Figure 5:**
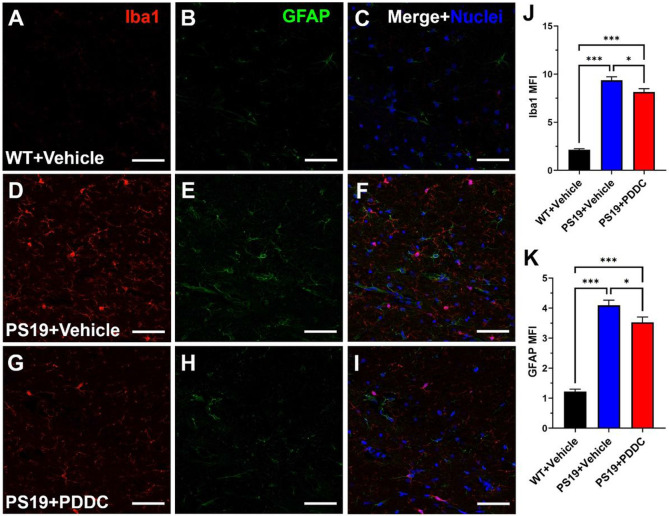
PDDC treatment reduces glial activation in the hippocampus of PS19 mice. Representative images from WT mice (**A-C**), vehicle-treated PS19 mice (**D-F**), and PDDC-treated PS19 mice (**G-I**). Microglia were stained with Iba1 (red). Astrocytes were stained with GFAP (green). Nuclei shown in blue. **J)** Quantification of Iba1 MFI staining. **K**) Quantification of GFAP MFI staining. N=216-232 images/group from 9-10 mice/group. Bars represent mean±SEM. **p<0.01, ***p<0.001. Scale bar, 50μm. Gamma and brightness adjusted equally for all images. One-way ANOVA with Tukey’s multiple comparison.

We previously reported that PDDC reduced EV release from astrocytes and activated microglia into plasma in a brain injury model(18, 33). Given that glial overactivation has been observed in PS19 mice(34), we sought to evaluate the effect of PDDC treatment. Microglial Iba1 MFI staining was elevated in PS19 mice compared to WT mice (Fig. 5A, D, J, p < 1.0×10^−15^). PDDC treatment in PS19 mice reduced Iba1 staining compared to vehicle-treated mice (Fig. 5D, G, J, p = 0.0148). Similarly, astrocyte GFAP MFI staining was elevated in PS19 versus WT mice (Fig. 5B, E, K, p < 1.0×10^−15^). PDDC treatment in the PS19 mice reduced the GFAP staining compared to vehicle-treated mice (Fig. 5E, H, K, p = 0.0298).

### PDDC treatment reduces the number of neuron- and microglia-derived EVs and their tau content in the plasma of PS19 mice

**Figure 6:**
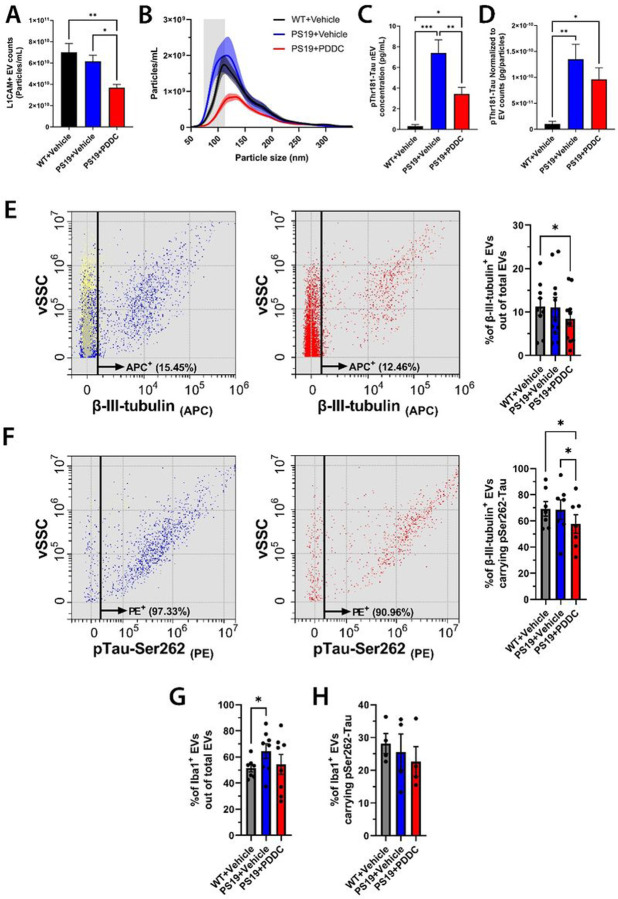
PDDC reduces plasma nEVs carrying pThr181-Tau in PS19 mice. **A**) Quantification of L1CAM+ nEVs immunocaptured from the plasma of WT mice, vehicle- and PDDC-treated PS19 mice by NTA. N=15-16. **B**) Averaged size profiles of L1CAM+ nEVs from the plasma of WT mice, vehicle- and PDDC-treated PS19 mice (N=15-16). **C**) pThr181-Tau in lysed L1CAM+ nEVs from WT mice, vehicle- and PDDC-treated PS19 mice. N=11-12. **D**) pThr181-Tau normalized to nEV concentration from WT mice, vehicle- and PDDC-treated PS19 mice. N=11-12. One-way ANOVA with Tukey’s multiple comparison. **E)** Dot plots showing the vSSC vs. APC-β-III-tubulin signal of BSE+ events gated in **Fig S5** for vehicle (left, blue events) and PDDC (middle, red events). Black line: threshold for APC-β-III-tubulin + events. Yellow events indicate negative control EVs labeled with BSE only. Bar graph: average percentage of APC-β-III-tubulin+ events out of total BSE+ events. **F)** Dot plots showing the vSSC vs PE-pTau-Ser262 signal of APC-β-III-tubulin+ events gated in **B**. Black line: threshold for PE-pTau-Ser262+ signal. Bar graph: average percentage of APC-β-III-tubulin+ events double-positive for PE-pTau-Ser262. **G and H)**Bar graph: mean percentage of APC-Iba-1+ events out of total BSE+ events (**G**) or APC-Iba-1+ events double-positive for PE-pTau-Ser262 (**H**) for each group. **E-H,** Two-way ANOVA. Bars represent mean±SEM. *p<0.05. **p<0.01. ***p<0.001.

Studies leveraging plasma samples from large longitudinal aging studies have found pTau cargo in neuron-derived EVs (nEVs) is a prognostic indicator of cognitive decline and AD diagnosis(52, 53). Therefore, we sought to evaluate the effects of PDDC on the number, size, and tau content of nEVs isolated from the plasma of PS19 mice. Flow cytometry analysis (FCA) at the single-EV level and ExoView confirmed that L1CAM immunocapture resulted in the recovery of detergent-sensitive membranous nanoparticles carrying canonical transmembrane and intravesicular EV markers (Fig. S4). Nanoparticle tracking analysis (NTA) revealed that PDDC-treated PS19 mice had reduced plasma nEV concentration when compared to both WT and vehicle-treated PS19 mice (Fig. 6A, p = 0.001 and p = 0.0187, respectively), mainly driven by a decrease of small EVs (< 150nm diameter*)(*Fig. 6B). We found higher levels of pThr181-Tau in lysed nEVs isolated from vehicle-treated PS19 mice compared to WT controls (Fig. 6C, p = 5.57×10^−6^), whereas PDDC treatment in PS19 mice exhibited reduced nEV pThr181-Tau (p = 0.0061). After normalization by L1CAM + EV concentration, pThr181-Tau increased in both vehicle- and PDDC-treated PS19 mice compared to WT (p = 0.0016 and p = 0.033, respectively), with a trend towards reduction with PDDC compared to vehicle (Fig. 6D).

At the single-EV level via FCA, the concentration of total plasma EVs identified using the fluorescent EV marker blue succinimidyl ester (BSE) remained unchanged in all treatment groups (Fig S5). PDDC-treated PS19 mice had decreased nEV percentages, identified using the neuron-specific marker β-III-tubulin, compared to both WT and vehicle-treated PS19 mice (Fig. 6E, p = 0.041), consistent with NTA findings for immunocaptured L1CAM + nEVs. PDDC treatment also reduced β-III-tubulin + nEVs carrying pSer262-Tau compared to vehicle-treated WT and PS19 mice (Fig. 6F, p = 0.022 and p = 0.027, respectively). FCA was also used to assess PDDC effects on EVs expressing Iba-1, a subpopulation enriched in microglia-derived EVs (mEVs). Vehicle-treated PS19 mice exhibited a higher percentage of Iba-1 + EVs compared to WT controls (p = 0.025) that trended lower with PDDC (Fig. 6G). We also observed a trend for PDDC-mediated reduction of Iba-1 + EVs carrying pSer262-Tau (Fig. 6H).

### PDDC treatment reduces tau spread in an AAV mutant hTau propagation model

**Figure 7:**
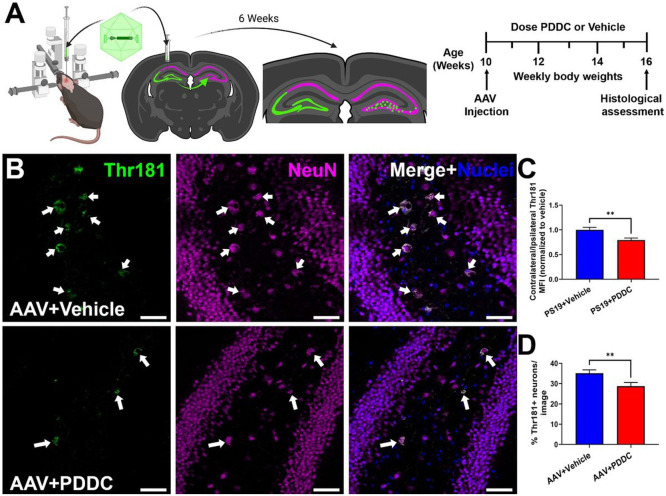
PDDC treatment reduces tau spread in an AAV mutant hTau propagation model. **A**) AAV-hTau model and dosing schematic. Mice were stereotaxically injected at 10 weeks-old into the left dorsal hippocampus with AAV-CBA-hTau24(P301L)(S320F)-WPRE which was taken up and expressed by cells in the left CA3 and dentate gyrus and propagated to the right DG hilus region over the course of 6 weeks. **B**) Representative images of the contralateral DG showing pThr181-Tau staining (green) from vehicle-treated (top) and PDDC-treated (bottom) AAV-hTau mice. Neurons stained with NeuN (magenta). Nuclei shown in blue. Scale bar, 50μm. Gamma and brightness adjusted equally for all images presented. **C**) Quantification of pThr181-Tau MFI of the contralateral DG normalized to the ipsilateral DG pThr181-Tau MFI. N=81-84 images/group from 17 mice/group. **D**) Quantification of the percentage of pThr181-Tau+ neurons in the contralateral dentate gyrus. N=56-72 images/group from 17 mice/group. Unpaired two-tailed t-test. **p<0.01. Bars represent mean±SEM.

Building upon similar models(16, 32, 54), our group developed a rapid tau propagation model where an AAV vector expressing double mutant hTau (P301L/S320F) is unilaterally injected into the hippocampus and the propagation of the hTau to the contralateral hippocampus is monitored over 6 weeks(15), during which time animals were treated with either vehicle or PDDC containing chow (Fig. 7A). PDDC-treated PS19 mice exhibited a significant reduction in contralateral pThr181-Tau MFI compared to vehicle-treated mice (Fig. 7B-C, p = 0.0052). There were also significantly fewer NeuN positive neurons in the contralateral hippocampus with pThr181-Tau expression (Fig. 7B, D, p = 0.0096). Ceramides were also altered with PDDC treatment. Of the 46 ceramides detected, 4 were significantly reduced in the hippocampus of PDDC treated mice compared to vehicle (Fig S6, Table S5, p values in table). Ceramide levels with PDDC treatment were similar to unaffected cortical levels agreeing with our prior study where nSMase2 activity levels were selectively elevated in the tau-expressing hippocampus but not the tau-naïve cortex(15).

## DISCUSSION

Herein we demonstrate that the potent and selective nSMase2 inhibitor PDDC chronically administered to PS19 mice is well-tolerated and can achieve sustained levels of the drug in the brain at concentrations capable of nSMase2 inhibition. Like human AD patients(20-25), we show, both *in vitro* and *in vivo*, that neurons expressing mutant human tau exhibit elevated ceramide levels. The tau-expressing hippocampi of both the PS19 and a AAV-hTau mice had elevated levels of brain nSMase2 enzymatic activity and ceramides. Both of these elevations were normalized by PDDC treatment, confirming brain target engagement. Also similar to human AD(26-28), PS19 mice exhibited elevated brain tau, thinning of the hippocampal neuronal cell layers, decreased mossy fiber synaptophysin staining, and elevated glial activation which were improved with PDDC. Additionally, PS19 mice exhibited increased tau content in their circulating plasma nEVs which was reduced with PDDC treatment. This is translationally exciting as studies leveraging plasma samples from large longitudinal studies of aging have shown that pTau cargo in nEVs can predict future cognitive decline in AD(52, 53). And lastly, using a newly developed AAV hTau injection propagation model, we directly demonstrated that PDDC treatment inhibited the spread of mutant hTau between synaptically connected brain regions.

In addition to their role in EV biogenesis(55), ceramides are elevated in AD patient brain tissue(20, 24, 25), plasma(22, 23), and CSF(21) and have been hypothesized to dysregulate autophagy, impair mitochondrial health, and induce senescence(reviewed in (56)). Here we show, for the first time, that several long and very long chain ceramides are significantly elevated in cultured neurons expressing mutant human tau, PS19 mouse brain, and specifically in the tau-expressing hippocampus of AAV-hTau mice. Treatment with PDDC reduced all aberrant ceramide species and in the majority of cases, completely normalized ceramides to WT baseline levels.

Ceramides are critical for EV biogenesis, as their enrichment at the plasma membrane enhances curvature and induces eventual EV budding(12). EVs are thought to play a role in the pathological spread of tau throughout the AD brain in a predictable manner along connectivity pathways in a prion-like fashion(3), with mounting evidence correlating tau propagation with disease progression(57, 58). The data observed from both the PS19 transgenic and AAV tau propagation models show that PDDC reduces tau spread by decreasing the absolute number of neurons that are seeded with pathological tau. We observed a significant reduction in the number of neurons that were tau positive in the contralateral DG in the mice injected with AAV-hTau following PDDC-versus vehicle-treatment. In the PS19 mice, while most hippocampal neurons had some tau expression, the intensity of the tau fluorescence measured individually within the neurons was significantly reduced in PDDC mice. These data suggest that PDDC did not alter the basal rate of tau accumulation driven by the neuronal promoter but, was inhibiting an alternative mechanism responsible for additional accumulation of tau in neurons, which we are proposing to be driven by EVs. Together, these data suggest that nSMase2 inhibition reduces hippocampal tau burden by slowing tau propagation by reducing the production of tau-containing EVs.

This hypothesis is further supported by our assessment of nEVs isolated from the plasma of PS19 mice. Biomarker studies in humans have shown that pThr181-Tau and pThr231-Tau in plasma nEVs predict AD diagnosis(53). Similar to AD patients, we found that pThr181-Tau levels in plasma nEVs were increased in PS19 mice. Chronic PDDC treatment could reduce this increase. In addition, PDDC decreased the number of nEVs in plasma, especially smaller nEVs. Using FCA, we observed a significant reduction in the percentage of nEVs that contained pSer262-Tau, at the single EV level. Taken together, these data demonstrate that PDDC treatment results in fewer nEVs circulating and, within this diminished population, a lower percentage of nEVs carrying pTau, thus reducing the overall tau seeding potential. This is therapeutically important as recent evidence suggests that seeding is likely the rate limiting step in AD disease progression(59). Reducing the number of tau-seeds via inhibiting the formation and/or spread of tau-carrying EVs represents a novel and to date unexplored therapeutic avenue for AD.

In addition to tau accumulation, PS19 mice showed synaptic loss and thinning of hippocampal cellular layers(34, 43, 60, 61). Importantly, these are pathological features also observed in human AD(26-28). PDDC improved the cell layer thickness of both the CA1 and dentate gyrus regions as well as increased the synaptophysin staining of the mossy fiber layer of the CA3 in PS19 mice. Since tau aggregation correlates with AD disease progression(57, 58) and studies utilizing human iPSC derived neurons show that tangle formation precedes neuronal death(62), reducing tau propagation via PDDC may meaningfully attenuate tau aggregation and delay neurotoxicity, contributing to improved cell layer thickness and maintenance of mossy fibers. These results suggest that by decreasing the spread of tau, PDDC also affords neuroprotective benefits.

Microglia and astrocytes have also been implicated in neuronal cell loss observed in AD (see review(63). Because both astrocyte-(64) and microglia-derived(16) EVs carry tau, and cortical glial reactivity positively correlates with AD neurofibrillary tangle burden(65), it is reasonable to suggest that at least some of the glial-mediated pathological contributions in AD stem from their role in the spread of tau. In fact, previous studies in both a rapid tau propagation model and the PS19 model showed that microglial depletion reduces tau burden(16). We therefore hypothesized that PDDC’s therapeutic benefit could, in part, be due to its impact on glia. Here we report that PDDC inhibited glial activation in PS19 mice. Specifically, we observed reduced intensity of both Iba1 and GFAP hippocampal staining. Moreover, we observed trends for PDDC reducing the percentage of plasma Iba-1 + EVs and the percentage of Iba-1 + EVs that are double-positive for PE-pTau-Ser262. These findings are in line with prior studies in an acute brain injury model showing that PDDC reduces the release of EVs into the systemic circulation from CD11b + activated microglia(33).

## CONCLUSIONS

The present study demonstrates that PDDC has multifaceted effects on the pathology of AD. This potent and selective nSMase2 inhibitor not only directly reduced tau propagation, but also decreased hippocampal gliosis, neuronal and synaptic degeneration and normalized brain ceramide levels. From a therapeutic perspective, this is exciting as these abnormalities have all been described in human AD. PDDC also reduced the number and absolute pTau levels in nEVs found in PS19 mouse plasma. Again, from a therapeutic perspective, this is important as increased tau in nEV in AD patients has been shown to positively correlate with disease progression. In summary, PDDC is a promising new therapeutic with the potential to slow the progression of AD by reducing the spread of hyperphosphorylated tau species.

## Supplementary Material

Supplement 1

## ABBREVIATIONS

AD: Alzheimer’s disease
Aβ: amyloid-beta
pTau: hyperphosphorylated tau
EVs: extracellular vesicles
nSMase2: neutral sphingomyelinase 2
PDDC: phenyl(R)-(1-(3-(3,4-dimethoxyphenyl)-2,6-dimethylimidazo[1,2-b]pyridazin-8-yl)pyrrolidin-3-yl)-carbamate
AAV: adeno-associated virus
MOI: multiplicity of infection
PFA: paraformaldehyde
SM: sphingomyelin
nEV: plasma neuronal EV
FCA: flow cytometry analysis
NTA: nanoparticle tracking analysis
Ctrl: control
MFI: mean fluorescence intensity
