# Supplementary material for: Inhibiting tau-induced elevated nSMase2 activity and ceramides is therapeutic in murine Alzheimer’s disease
Source: Res Sq. 2023 Jul 18:rs.3.rs-3131295. Preprint. [Version 1] doi: 10.21203/rs.3.rs-3131295/v1 (PMC10371082; doi:10.21203/rs.3.rs-3131295/v1)
Supplement: Supplement 1 [file NIHPPrs3131295v1-supplement-1.pdf]

SUPPLEMENTARY REFERENCES

1. Biogen. Biogen and Eisai Announce Design of ADUHELM ICARE AD-US Study, the First Real-World Observational Phase 4 Study in Alzheimer’s Disease at AAIC 2021. 2021.
2. Avgerinos KI, Ferrucci L, Kapogiannis D. Effects of monoclonal antibodies against amyloid-beta on clinical and biomarker outcomes and adverse event risks: A systematic review and meta-analysis of phase III RCTs in Alzheimer's disease. Ageing Res Rev. 2021,68:101339.
3. Braak H, Braak E. Neuropathological stageing of Alzheimer-related changes. Acta Neuropathologica. 1991,82(4):239-59.
4. Ossenkoppele R, Pichet Binette A, Groot C, Smith R, Strandberg O, Palmqvist S, et al. Amyloid and tau PET-positive cognitively unimpaired individuals are at high risk for future cognitive decline. Nature medicine. 2022,28(11):2381-7.
5. Simon D, Garcia-Garcia E, Royo F, Falcon-Perez JM, Avila J. Proteostasis of tau. Tau overexpression results in its secretion via membrane vesicles. FEBS Lett. 2012,586(1):47-54.
6. Gibbons GS, Lee VMY, Trojanowski JQ. Mechanisms of Cell-to-Cell Transmission of Pathological Tau: A Review. JAMA Neurol. 2019,76(1):101-8.
7. Yanamandra K, Jiang H, Mahan TE, Maloney SE, Wozniak DF, Diamond MI, et al. Anti-tau antibody reduces insoluble tau and decreases brain atrophy. Annals of clinical and translational neurology. 2015,2(3):278-88.
8. Mullard A. Failure of first anti-tau antibody in Alzheimer disease highlights risks of history repeating. Nat Rev Drug Discov. 2021,20(1):3-5.
9. Imbimbo BP, Balducci C, Ippati S, Watling M. Initial failures of anti-tau antibodies in Alzheimer's disease are reminiscent of the amyloid-beta story. Neural Regen Res. 2023,18(1):117-8.
10. Saman S, Kim W, Raya M, Visnick Y, Miro S, Saman S, et al. Exosome-associated tau is secreted in tauopathy models and is selectively phosphorylated in cerebrospinal fluid in early Alzheimer disease. J Biol Chem. 2012,287(6):3842-9.
11. Ruan Z, Pathak D, Venkatesan Kalavai S, Yoshii-Kitahara A, Muraoka S, Bhatt N, et al. Alzheimer's disease brain-derived extracellular vesicles spread tau pathology in interneurons. Brain. 2021,144(1):288-309.
12. Trajkovic K, Hsu C, Chiantia S, Rajendran L, Wenzel D, Wieland F, et al. Ceramide triggers budding of exosome vesicles into multivesicular endosomes. Science. 2008,319(5867):1244-7.
13. Tallon C, Hollinger KR, Pal A, Bell BJ, Rais R, Tsukamoto T, et al. Nipping disease in the bud: nSMase2 inhibitors as therapeutics in extracellular vesicle-mediated diseases. Drug Discovery Today. 2021.
14. Tabatadze N, Savonenko A, Song H, Bandaru VV, Chu M, Haughey NJ. Inhibition of neutral sphingomyelinase-2 perturbs brain sphingolipid balance and spatial memory in mice. Journal of

neuroscience research. 2010,88(13):2940-51.

15. Tallon C, Bell BJ, Sharma A, Pal A, Malvankar MM, Thomas AG, et al. Dendrimer-Conjugated nSMase2 Inhibitor Reduces Tau Propagation in Mice. Pharmaceutics. 2022,14(10).
16. Asai H, Ikezu S, Tsunoda S, Medalla M, Luebke J, Haydar T, et al. Depletion of microglia and inhibition of exosome synthesis halt tau propagation. Nat Neurosci. 2015,18(11):1584-93.
17. Bilousova T, Simmons BJ, Knapp RR, Elias CJ, Campagna J, Melnik M, et al. Dual Neutral Sphingomyelinase-2/Acetylcholinesterase Inhibitors for the treatment of Alzheimer's disease. ACS Chemical Biology. 2020.
18. Rojas C, Sala M, Thomas AG, Datta Chaudhuri A, Yoo SW, Li Z, et al. A novel and potent brain penetrant inhibitor of extracellular vesicle release. Br J Pharmacol. 2019,176(19):3857-70.
19. Sala M, Hollinger KR, Thomas AG, Dash RP, Tallon C, Veeravalli V, et al. Novel human neutral sphingomyelinase 2 inhibitors as potential therapeutics for Alzheimer disease. Journal of Medicinal Chemistry. 2020.
20. Filippov V, Song MA, Zhang K, Vinters HV, Tung S, Kirsch WM, et al. Increased ceramide in brains with Alzheimer's and other neurodegenerative diseases. J Alzheimers Dis. 2012,29(3):537-47.
21. Teitsdottir UD, Halldorsson S, Rolfsson O, Lund SH, Jonsdottir MK, Snaedal J, et al. Cerebrospinal Fluid C18 Ceramide Associates with Markers of Alzheimer's Disease and Inflammation at the Pre- and Early Stages of Dementia. J Alzheimers Dis. 2021,81(1):231-44.
22. Mielke MM, Haughey NJ, Ratnam Bandaru VV, Schech S, Carrick R, Carlson MC, et al. Plasma ceramides are altered in mild cognitive impairment and predict cognitive decline and hippocampal volume loss. Alzheimers Dement. 2010,6(5):378-85.
23. Mielke MM, Haughey NJ, Han D, An Y, Bandaru VVR, Lyketsos CG, et al. The Association Between Plasma Ceramides and Sphingomyelins and Risk of Alzheimer's Disease Differs by Sex and APOE in the Baltimore Longitudinal Study of Aging. J Alzheimers Dis. 2017,60(3):819-28.
24. Satoi H, Tomimoto H, Ohtani R, Kitano T, Kondo T, Watanabe M, et al. Astroglial expression of ceramide in Alzheimer's disease brains: a role during neuronal apoptosis. Neuroscience. 2005,130(3):657-66.
25. Cutler RG, Pedersen WA, Camandola S, Rothstein JD, Mattson MP. Evidence that accumulation of ceramides and cholesterol esters mediates oxidative stress-induced death of motor neurons in amyotrophic lateral sclerosis. Ann Neurol. 2002,52(4):448-57.
26. Terry RD, Masliah E, Salmon DP, Butters N, DeTeresa R, Hill R, et al. Physical basis of cognitive alterations in alzheimer's disease: Synapse loss is the major correlate of cognitive impairment. Annals of Neurology. 1991,30(4):572-80.
27. West MJ, Coleman PD, Flood DG, Troncoso JC. Differences in the pattern of hippocampal neuronal loss in normal ageing and Alzheimer's disease. The Lancet. 1994,344(8925):769-72.
28. Gemmell E, Bosomworth H, Allan L, Hall R, Khundakar A, Oakley AE, et al. Hippocampal Neuronal Atrophy and Cognitive Function in Delayed Poststroke and Aging-Related Dementias. Stroke. 2012,43(3):808-14.

29. Gao R-D, Maeda M, Tallon C, Feinberg AP, Slusher BS, Tsukamoto T. Effects of 6-Aminonicotinic Acid Esters on the Reprogrammed Epigenetic State of Distant Metastatic Pancreatic Carcinoma. ACS Med Chem Lett. 2022,13(12):1892-7.
30. Yoo SW, Bae M, Tovar-Y-Romo LB, Haughey NJ. Hippocampal encoding of interoceptive context during fear conditioning. Translational psychiatry. 2017,7(1):e991-e.
31. Wheeler D, Knapp E, Bandaru VV, Wang Y, Knorr D, Poirier C, et al. Tumor necrosis factor-alpha-induced neutral sphingomyelinase-2 modulates synaptic plasticity by controlling the membrane insertion of NMDA receptors. J Neurochem. 2009,109(5):1237-49.
32. Koller EJ, Gonzalez De La Cruz E, Machula T, Ibanez KR, Lin W-L, Williams T, et al. Combining P301L and S320F tau variants produces a novel accelerated model of tauopathy. Human Molecular Genetics. 2019,28(19):3255-69.
33. Tallon C, Picciolini S, Yoo S-W, Thomas AG, Pal A, Alt J, et al. Inhibition of neutral sphingomyelinase 2 reduces extracellular vesicle release from neurons, oligodendrocytes, and activated microglial cells following acute brain injury. Biochemical Pharmacology. 2021:114796.
34. Yoshiyama Y, Higuchi M, Zhang B, Huang SM, Iwata N, Saido TC, et al. Synapse loss and microglial activation precede tangles in a P301S tauopathy mouse model. Neuron. 2007,53(3):337-51.
35. Rojas C, Barnaeva E, Thomas AG, Hu X, Southall N, Marugan J, et al. DPTIP, a newly identified potent brain penetrant neutral sphingomyelinase 2 inhibitor, regulates astrocyte-peripheral immune communication following brain inflammation. Sci Rep. 2018,8(1):17715.
36. Figuera-Losada M, Stathis M, Dorskind JM, Thomas AG, Bandaru VV, Yoo SW, et al. Cambinol, a novel inhibitor of neutral sphingomyelinase 2 shows neuroprotective properties. PLoS One. 2015,10(5):e0124481.
37. Bligh EG, Dyer WJ. A rapid method of total lipid extraction and purification. Canadian journal of biochemistry and physiology. 1959,37(8):911-7.
38. Zhu X, Hollinger KR, Huang Y, Borjabad A, Kim BH, Arab T, et al. Neutral sphingomyelinase 2 inhibition attenuates extracellular vesicle release and improves neurobehavioral deficits in murine HIV. Neurobiol Dis. 2022,169:105734.
39. Haughey NJ, Cutler RG, Tamara A, McArthur JC, Vargas DL, Pardo CA, et al. Perturbation of sphingolipid metabolism and ceramide production in HIV-dementia. Ann Neurol. 2004,55(2):257-67.
40. Bandaru VV, McArthur JC, Sacktor N, Cutler RG, Knapp EL, Mattson MP, et al. Associative and predictive biomarkers of dementia in HIV-1-infected patients. Neurology. 2007,68(18):1481-7.
41. Sahara N, Kimura T. Biochemical Properties of Pathology-Related Tau Species in Tauopathy Brains: An Extraction Protocol for Tau Oligomers and Aggregates. In: Sigurdsson EM, Calero M, Gasset M, editors. Amyloid Proteins: Methods and Protocols. New York, NY: Springer New York, 2018. p. 435-45.
42. Hollinger KR, Sharma A, Tallon C, Lovell L, Thomas AG, Zhu X, et al. Dendrimer-2PMPA selectively blocks upregulated microglial GCPII activity and improves cognition in a mouse model of multiple sclerosis. Nanotheranostics. 2022,6(2):126-42.

43. Gratuze M, Leyns CEG, Sauerbeck AD, St-Pierre M-K, Xiong M, Kim N, et al. Impact of TREM2R47H variant on tau pathology–induced gliosis and neurodegeneration. The Journal of Clinical Investigation. 2020,130(9):4954-68.
44. Delgado-Peraza F, Nogueras-Ortiz CJ, Volpert O, Liu D, Goetzl EJ, Mattson MP, et al. Neuronal and Astrocytic Extracellular Vesicle Biomarkers in Blood Reflect Brain Pathology in Mouse Models of Alzheimer's Disease. Cells. 2021,10(5).
45. Gomes PA, Bodo C, Nogueras-Ortiz C, Samiotaki M, Chen M, Soares-Cunha C, et al. A novel isolation method for spontaneously released extracellular vesicles from brain tissue and its implications for stress-driven brain pathology. Cell Commun Signal. 2023,21(1):35.
46. Patel H, Martinez P, Perkins A, Taylor X, Jury N, McKinzie D, et al. Pathological tau and reactive astrogliosis are associated with distinct functional deficits in a mouse model of tauopathy. Neurobiology of Aging. 2022,109:52-63.
47. Simon MM, Greenaway S, White JK, Fuchs H, Gailus-Durner V, Wells S, et al. A comparative phenotypic and genomic analysis of C57BL/6J and C57BL/6N mouse strains. Genome biology. 2013,14(7):R82.
48. Otto GP, Rathkolb B, Oestereicher MA, Lengger CJ, Moerth C, Micklich K, et al. Clinical Chemistry Reference Intervals for C57BL/6J, C57BL/6N, and C3HeB/FeJ Mice (Mus musculus). Journal of the American Association for Laboratory Animal Science : JAALAS. 2016,55(4):375-86.
49. Laboratory TJ. Physiological Data Summary - C57BL/6J (000664) [Available from: https://jackson.jax.org/rs/444-BUH-304/images/physiological_data_000664.pdf.
50. Charles River Larboratories International I. C57BL/6 Mice Datasheet 2019 [Available from: https://www.criver.com/sites/default/files/resources/C57BL6MouseModelInformationSheet.pdf.
51. Taconic Biosciences I. Automated Clinical Chemistry Analysis (ACCA) 2011 [updated 12/19/2011. 12/19/2011:[Available from: https://www.taconic.com/phenotypic-data/automated-clinical-chemistry-analysis/.
52. Fiandaca MS, Kapogiannis D, Mapstone M, Boxer A, Eitan E, Schwartz JB, et al. Identification of preclinical Alzheimer's disease by a profile of pathogenic proteins in neurally derived blood exosomes: A case-control study. Alzheimers Dement. 2015,11(6):600-7.e1.
53. Kapogiannis D, Mustapic M, Shardell MD, Berkowitz ST, Diehl TC, Spangler RD, et al. Association of Extracellular Vesicle Biomarkers With Alzheimer Disease in the Baltimore Longitudinal Study of AgingAssociation of Extracellular Vesicle Biomarkers With Alzheimer DiseaseAssociation of Extracellular Vesicle Biomarkers With Alzheimer Disease. JAMA Neurology. 2019.
54. Bell BJ, Malvankar MM, Tallon C, Slusher BS. Sowing the Seeds of Discovery: Tau-Propagation Models of Alzheimer’s Disease. ACS Chemical Neuroscience. 2020.
55. Trajkovic K, Hsu C, Chiantia S, Rajendran L, Wenzel D, Wieland F, et al. Ceramide Triggers Budding of Exosome Vesicles into Multivesicular Endosomes. Science. 2008,319(5867):1244-7.
56. Chowdhury MR, Jin HK, Bae JS. Diverse Roles of Ceramide in the Progression and Pathogenesis of Alzheimer's Disease. Biomedicines. 2022,10(8).

57. Lowe VJ, Wiste HJ, Senjem ML, Weigand SD, Therneau TM, Boeve BF, et al. Widespread brain tau and its association with ageing, Braak stage and Alzheimer's dementia. Brain. 2018,141(1):271-87.
58. Ossenkoppele R, Schonhaut DR, Scholl M, Lockhart SN, Ayakta N, Baker SL, et al. Tau PET patterns mirror clinical and neuroanatomical variability in Alzheimer's disease. Brain. 2016,139(Pt 5):1551-67.
59. Meisl G, Hidari E, Allinson K, Rittman T, DeVos SL, Sanchez JS, et al. In vivo rate-determining steps of tau seed accumulation in Alzheimer&#x2019,s disease. Science Advances. 2021,7(44):eabh1448.
60. DeVos SL, Miller RL, Schoch KM, Holmes BB, Kebodeaux CS, Wegener AJ, et al. Tau reduction prevents neuronal loss and reverses pathological tau deposition and seeding in mice with tauopathy. Sci Transl Med. 2017,9(374).
61. Leyns CEG, Ulrich JD, Finn MB, Stewart FR, Koscal LJ, Remolina Serrano J, et al. TREM2 deficiency attenuates neuroinflammation and protects against neurodegeneration in a mouse model of tauopathy. Proc Natl Acad Sci U S A. 2017,114(43):11524-9.
62. Oakley DH, Klickstein N, Commins C, Chung M, Dujardin S, Bennett RE, et al. Continuous Monitoring of Tau-Induced Neurotoxicity in Patient-Derived iPSC-Neurons. The Journal of Neuroscience. 2021,41(19):4335.
63. Henstridge CM, Hyman BT, Spires-Jones TL. Beyond the neuron-cellular interactions early in Alzheimer disease pathogenesis. Nature reviews Neuroscience. 2019,20(2):94-108.
64. Goetzl EJ, Mustapic M, Kapogiannis D, Eitan E, Lobach IV, Goetzl L, et al. Cargo proteins of plasma astrocyte-derived exosomes in Alzheimer's disease. FASEB journal : official publication of the Federation of American Societies for Experimental Biology. 2016,30(11):3853-9.
65. Serrano-Pozo A, Mielke ML, Gómez-Isla T, Betensky RA, Growdon JH, Frosch MP, et al. Reactive glia not only associates with plaques but also parallels tangles in Alzheimer's disease. Am J Pathol. 2011,179(3):1373-84.
